# The Role of Exosomes in the Regulation of Molecular Mechanisms Underlying Treatment Resistance—Linking Cellular Crosstalk to Clinical Implications in Depression

**DOI:** 10.3390/ijms27052449

**Published:** 2026-03-06

**Authors:** Kinga Dyndał, Patrycja Pańczyszyn-Trzewik, Bernadetta Jakubowska, Magdalena Sowa-Kućma

**Affiliations:** 1 Student Scientific Club “NEURON”, Faculty of Medicine, Collegium Medicum, University of Rzeszów, Al. Tadeusza Rejtana 16C, 35-959 Rzeszów, Poland; kd121949@stud.ur.edu.pl (K.D.); bj117556@stud.ur.edu.pl (B.J.); 2 Department of Human Physiology, Faculty of Medicine, Collegium Medicum, University of Rzeszów, Al. Tadeusza Rejtana 16C, 35-959 Rzeszów, Poland; ppanczyszyn@ur.edu.pl; 3 Centre for Innovative Research in Medical and Natural Sciences, Faculty of Medicine, Collegium Medicum, University of Rzeszów, Warzywna 1a, 35-310 Rzeszów, Poland

**Keywords:** depressive disorders, TRD, exosomes, multi-omics, oxidative stress, neuroinflammation, neuroplasticity

## Abstract

Depressive disorders (DDs), especially treatment-resistant depression (TRD), pose a significant challenge worldwide, largely because their underlying biological mechanisms are complicated and treatments often fall short. There is growing evidence pointing to factors like disrupted neuroplasticity, neuroinflammation, irregularities in the hypothalamic–pituitary–adrenal (HPA) axis, and glutamatergic system imbalances as contributors to the onset and persistence of depressive symptoms. Exosomes (small extracellular vesicles involved in communication between cells) have recently gained attention for their potential role in connecting peripheral and central nervous system (CNS) changes. They carry proteins, lipids, and nucleic acids and are even capable of crossing the blood–brain barrier. Because of this, exosomes might provide a window into molecular changes in the brain and serve as accessible biomarkers of disease status and treatment response. Recent research points out that the contents of exosomes, especially microRNAs (miRNAs) and neurotrophic factors like brain-derived neurotrophic factor (BDNF), might play a part in disrupting synaptic plasticity and could be linked to resistance to antidepressants. At the same time, there is growing interest in using engineered exosomes as targeted drug carriers aimed at the CNS. That said, there are still quite a few hurdles to overcome. Methods vary widely between studies, protocols for isolating exosomes are not sufficiently standardized, safety data are limited, and we do not fully understand how drugs and exosomes interact or how they behave pharmacokinetically. This review brings together current findings regarding exosomes in DDs (with particular emphasis on TRD), highlights their promise for diagnosis and treatment, and sets out some of the main questions that need to be answered before clinical application becomes feasible.

## 1. Introduction

Depressive disorders (DDs) are among the most prevalent and disabling neuropsychiatric conditions worldwide, affecting more than 280 million individuals and constituting a leading cause of global disability [1]. Beyond the profound psychological burden, they are associated with increased morbidity and mortality, substantial socioeconomic costs, and a marked deterioration in quality of life [2].

Major Depressive Disorder (MDD), commonly referred to as clinical depression, represents the most prevalent and clinically significant form within DDs. It is characterized by persistent low mood, anhedonia, cognitive and psychomotor disturbances, and functional impairment lasting at least two weeks. Among its most severe manifestations is suicidal ideation, which significantly increases the risk of suicide attempts and completed suicide [3]. Importantly, MDD is not a homogeneous condition; rather, it encompasses a spectrum of clinical trajectories, symptom profiles, and treatment responses. First-line treatment of MDD primarily relies on pharmacotherapies targeting monoaminergic (particularly serotonergic, noradrenergic, and dopaminergic) neurotransmission [4]. Although these agents are effective for many patients, a substantial proportion fail to achieve sustained remission. Approximately 30% of individuals with MDD meet criteria for treatment-resistant depression (TRD) [5]. According to the European Medicines Agency and the World Federation of Societies of Biological Psychiatry (WFSBP), TRD is typically defined as the absence of a clinically meaningful response following at least two adequate trials of antidepressants from different pharmacological classes, administered at appropriate doses and durations with confirmed adherence [6]. In line with contemporary classifications (including ICD-11 and DSM-5-TR), TRD is not recognized as a distinct diagnostic entity but rather conceptualized as a subtype or stage of MDD reflecting insufficient response to standard treatment [7].

However, accumulating molecular and neurobiological evidence suggests that TRD may represent more than a purely clinical categorization. Increasing data support the notion of TRD as a biologically distinct phenotype within the depressive spectrum [8]. Alterations in glutamatergic neurotransmission are particularly prominent. Dysregulation of N-methyl-D-aspartate receptor (NMDAR) subunits (notably GluN1 and GluN2B) and impaired α-amino-3-hydroxy-5-methyl-4-isoxazolepropionic acid receptor (AMPAR) trafficking contribute to disrupted synaptic plasticity [9]. The rapid and robust antidepressant effects of ketamine (an NMDAR antagonist) further underscore the central role of glutamatergic signaling in the pathophysiology of TRD. Importantly, its clinical relevance is reinforced by regulatory approval (FDA has approved the intranasal formulation of esketamine for TRD), making glutamatergic modulation not only a mechanistic hypothesis but also an established and clinically available therapeutic strategy [10].

Impaired neuroplasticity in TRD is also associated with reduced levels of neurotrophic factors, including brain-derived neurotrophic factor (BDNF) and vascular endothelial growth factor (VEGF) [11,12]. These alterations often coexist with chronic neuroinflammation, sustained psychosocial stress, and increased oxidative stress, e.g., processes that appear more pronounced in TRD than in treatment-responsive depression [13,14]. Elevated concentrations of proinflammatory cytokines such as IL-6, IL-8, and TNF-α may disrupt hypothalamic–pituitary–adrenal (HPA) axis regulation, thereby impairing glucocorticoid receptor signaling and leading to persistent hypercortisolemia [15]. Chronic HPA axis activation can, in turn, stimulate microglial reactivity, leading to excessive production of reactive oxygen species (ROS) and reactive nitrogen species (RNS), neuronal damage, and further compromise of synaptic integrity [13].

Genetic vulnerability also appears to modulate treatment response. Studies indicate that the aforementioned *GRIN2B* gene has been identified as a potential predictor of TRD in patients with MDD, while IL6R polymorphisms may influence the response to pharmacotherapy in severe depression [16,17]. Neuroimaging findings complement these molecular observations. Patients with TRD exhibit a wide spectrum of structural and functional changes within the cortico-limbic network, which will be discussed later in this article [18,19].

Taken together, these findings suggest that TRD extends beyond a simple lack of therapeutic response. Instead, it reflects a complex interplay of maladaptive neuroplasticity, persistent neuroinflammation, stress-axis dysregulation, and large-scale network disorganization. At the same time, no single molecular pathway has been identified as uniquely and exclusively altered in TRD [9,10,11,12,13,14,15,16,17,18,19]. Rather, treatment resistance likely arises from quantitative and qualitative differences in the severity, persistence, and interactions among dysregulated biological systems. This heterogeneity highlights the limitations of current monoamine-based strategies and underscores the urgent need for reliable biomarkers and novel mechanistic targets.

In recent years, growing attention has focused on exosomes (EXOs) as potential mediators and indicators of these pathological processes. EXOs are nanosized extracellular vesicles released by nearly all cell types under both physiological and pathological conditions [20]. They constitute an additional layer of intercellular communication, complementing classical synaptic and humoral signaling. EXOs transport diverse molecular cargo (including microRNAs-miRNAs, messenger RNAs, long non-coding RNAs, proteins, and lipids) that reflect the functional state of their cells of origin [21,22,23]. Importantly, their ability to cross the blood–brain barrier (BBB) has opened new possibilities for studying central nervous system (CNS) processes through minimally invasive peripheral sampling (e.g., blood or saliva) [24]. Emerging evidence indicates that altered exosomal cargo may be associated with TRD. For instance, dysregulated expression of specific miRNAs in plasma-derived EXOs (such as increased miR-335-5p and decreased miR-1292-3p) has been linked to pathways involved in neuroplasticity, neuroinflammation, and oxidative stress [25]. These findings suggest that exosome-mediated molecular signaling may contribute to persistent maladaptive cellular crosstalk, thereby sustaining treatment resistance.

From a clinical perspective, EXOs offer two particularly promising avenues. First, they may serve as minimally invasive biomarkers that can improve diagnostic stratification, identify high-risk patients, and predict therapeutic response. Second, they hold potential as therapeutic tools or delivery platforms, enabling targeted transport of bioactive molecules with anti-inflammatory, neuroprotective, or neuromodulatory properties. The integration of exosome-derived molecular signatures with multi-omics technologies and advanced computational approaches, including artificial intelligence, may further enhance patient stratification and facilitate personalized treatment strategies within the framework of precision psychiatry [26].

Given the limited understanding of molecular mechanisms underlying treatment resistance and the lack of reliable biomarkers guiding individualized therapy, there is a pressing need for novel approaches that integrate pathophysiological insights with translational potential. Exosomes, as key mediators of intercellular communication capable of crossing the blood–brain barrier, represent a promising link between peripheral molecular signatures and CNS pathology. This review aims to critically evaluate current evidence on the role of exosomes in DDs, with particular emphasis on TRD. We discuss their involvement in neuroplasticity, neuroinflammation, and glutamatergic dysregulation, assess their potential as diagnostic and predictive biomarkers, and explore emerging therapeutic strategies based on exosome engineering. Finally, we outline the major methodological and translational challenges that must be addressed to advance exosome-based approaches toward clinical implementation.

## 2. Methodology

We conducted a structured search of the literature using PubMed/MEDLINE, Scopus, Embase, Web of Science, and Google Scholar. The search strategy combined the following keywords: “depressive disorder,” “treatment-resistant depression,” “exosomes,” “biomarkers,” and “miRNA.” To complement the database search and capture ongoing or recently completed studies, the ClinicalTrials.gov registry was also reviewed. Our primary focus was on publications from 2020 to 2025 that explored exosome-derived biomarkers and their relevance to TRD.

In preparing this review, we examined both clinical and preclinical research, including animal and translational studies, that investigated the role of exosomes in depressive pathology. Particular attention was paid to their involvement in intercellular communication, neuroplasticity, metabolic regulation, and neuroinflammatory processes, as well as to recent developments in exosome-based diagnostic approaches and emerging therapeutic strategies.

**Figure 1:**
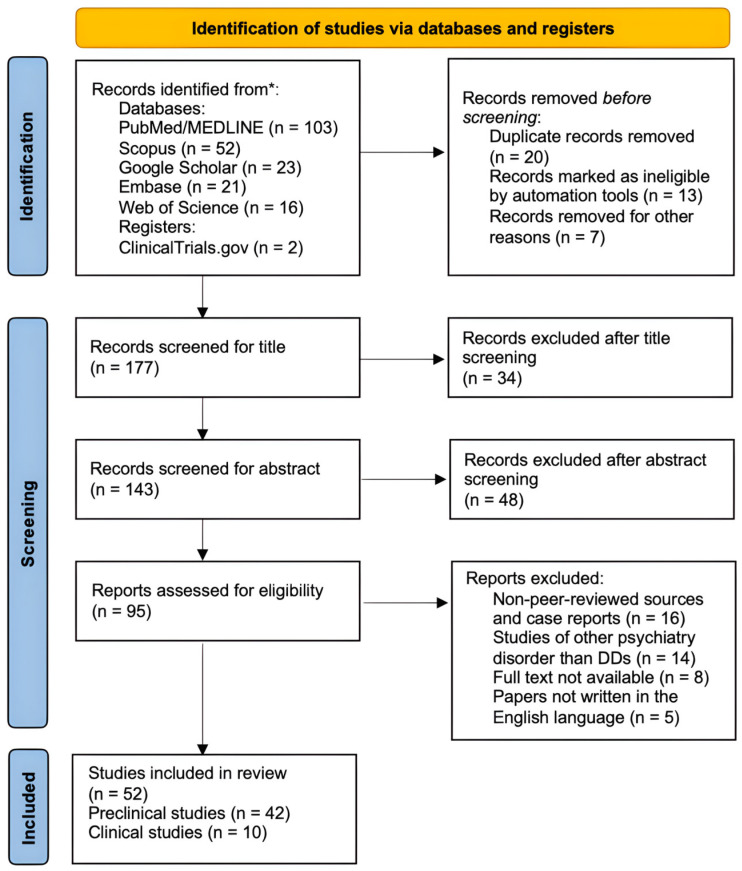
PRISMA flow diagram illustrating the study selection process. * the number of records identified from each database or register searched.

To maintain scientific rigor, we limited our analysis to peer-reviewed publications. Case reports and non-peer-reviewed sources were excluded. Studies focusing on psychiatric conditions other than DDs (such as bipolar disorder or schizophrenia) were not included unless they offered mechanistic insights directly applicable to TRD. The overall selection process is summarized in Figure 1.

For the sake of clarity and consistency, we use the term “EXOs” throughout this manuscript. At the same time, we acknowledge that the terminology in the field is not uniform. Depending on methodological approaches and classification criteria, different studies refer to these vesicles as extracellular vesicles (EVs), small extracellular vesicles (sEVs), neuron-derived exosomes (NDEs), or other cell-specific subtypes. In this review, we retain the original terminology when discussing individual studies, but use “EXOs” as a unifying term where appropriate.

## 3. Neurobiological and Pathophysiological Mechanisms Underlying MDD and TRD

### 3.1. General Pathophysiological Features and Biomarker Perspectives in TRD

TRD remains a condition with a multifactorial and not yet fully understood pathophysiology [27]. Importantly, no specific markers for TRD have yet been identified, and the diagnosis itself is currently based on clinical criteria, which highlights the need for research aimed at identifying objective biological markers. Some of the dysregulated neurobiological pathways observed in MDD appear to be more pronounced or persistent in TRD, according to preclinical and clinical studies, which may potentially contribute to reduced treatment response [28].

It should be noted that the abnormalities observed in TRD may include chronic neuroinflammation and oxidative stress, dysregulation of the (HPA axis, and alterations in neurotransmitter systems. An important limitation in the study of TRD pathomechanisms is the lack of a specific animal model; attempts to create one have been unsuccessful. Currently, research on treatment resistance primarily uses classic, well-defined animal models of depression, such as chronic unpredictable mild stress (CUMS), chronic mild stress (CMS), and lipopolysaccharide (LPS)-induced models, to study treatment resistance. It is important to note that these models mainly show construct validity for depression-related traits, rather than for TRD as a separate clinical condition. Take the CUMS model, for instance; it effectively triggers anhedonia and dysregulation of the HPA axis, mimicking key symptoms of depression. Likewise, chronic social stress models) replicate stress vulnerability and behavioral despair, while LPS-based models highlight inflammation-driven depressive behaviors. However, even though these models might exhibit less responsiveness to long-term antidepressant treatment under certain experimental conditions, they do not consistently produce a stable phenotype of pharmacological non-response that mirrors clinical TRD. Recent analyses of preclinical TRD research, including a review by Kolasa and Faron-Górecka [29], emphasize that current models capture specific aspects of treatment resistance like ongoing anhedonia, neuroinflammatory activation, or impaired neuroplasticity, but they fall short of reflecting the complex and evolving nature of TRD seen in patients. Therefore, these models might be more indicative of a susceptibility to antidepressant non-response rather than a fully developed treatment-resistant phenotype. These models may reproduce certain aspects of antidepressant non-response; however, they do not fully capture the chronic, clinically defined, and treatment-refractory nature of TRD, as they predominantly reflect reversible, experimentally induced behavioral and neurobiological changes [30].

Taking into account the numerous limitations of research on the pathomechanisms of DDs, including TRD, increasing attention has recently been paid to EXOs, extracellular vesicles measuring 30–150 nm, which, due to their bilayer structure, can cross the BBB [31]. This property means that they may serve as an indirect source reflecting processes occurring in the CNS, while being detectable in peripheral biological materials [32]. Analysis of their content, including specific miRNAs, proteins, and lipids, may in the future enable the identification of potential biomarkers for TRD [33].

### 3.2. Neuroinflammation and HPA Axis Dysregulation

Neuroinflammation is defined as a condition characterized by abnormal activation of the immune response in the CNS, involving dysregulated activity of glial cells, primarily microglia and astrocytes, and the release of proinflammatory mediators, alongside elevated systemic markers such as CRP [34].

Neuroinflammation is increasingly recognized as a key component of depression pathophysiology, as supported by numerous preclinical studies employing animal models with experimentally induced inflammatory activation, including chronic stress paradigms (e.g., chronic social defeat stress—CSDS, CUMS, chronic restraint stress—CRS) and pharmacological induction (e.g., LPS) [35]. In fact, neuroinflammation is accompanied by a phenotypic change in microglia, characterized by morphological alterations and an increased release of pro-inflammatory cytokines (e.g., IL-6, IL-8, TNF-α, and IFN-γ), which may contribute to the development and maintenance of depressive pathology [34]. Depending on the MDD phenotype, specific inflammatory signaling molecules and alternative metabolic pathways may be differentially activated, shaping the course and intensity of neuroinflammatory processes. Importantly, accumulating evidence indicates that a subgroup of patients with TRD exhibits persistent low-grade inflammation, suggesting that immune dysregulation may contribute to treatment resistance [15]. For example, Duda et al. subjected rats to CMS procedure and demonstrated that approximately 20% of rats did not respond to imipramine treatment (10 mg/kg b.w.; CMS IMI-NR group), as evidenced by the persistence of anhedonia, one of the most important symptoms of TRD. Compared to the treatment-responsive rats (CMS IMI-R group), the CMS IMI-NR animals exhibited increased IL-6 mRNA expression in both the cortex and hippocampus. Additionally, CMS IMI-R rats showed significant reductions in IL-6 and IFN-γ levels. These findings indicate that persistent neuroinflammatory activation is associated with antidepressant nonresponse and may contribute to a TRD-like phenotype [36].

Patients with MDD exhibit elevated levels of inflammatory markers, including CRP, IL-6, and TNF-α [37]. Furthermore, studies indicate that increased levels of proinflammatory markers correlate with greater symptom severity [38,39]. However, a systematic review evaluating CRP as a biomarker in MDD demonstrated that low-grade inflammation is present in a subset of patients with reduced treatment responsiveness, suggesting a distinct etiopathogenesis [40]. In line with these findings, emerging evidence suggests that inflammatory activation is particularly pronounced in TRD, as patients with TRD demonstrate persistently higher levels of proinflammatory markers compared with treatment-responsive individuals. For example, Chamberlain et al. showed that peripheral CRP concentrations were significantly elevated in the treatment-resistant group compared with treatment-responsive and untreated groups [41]. Moreover, results published by Strawbridge et al. indicate that increased inflammatory proteins, such as IL-6 and TNF-α, in TRD patients were associated with poorer treatment response. Patients with higher levels of systemic inflammation tended to have greater symptom severity, cognitive impairment, and increased treatment resistance, highlighting the complex interaction between neuroinflammation, depression neurobiology, and clinical trajectory [42]. Notably, IL-6 signaling may be further enhanced by binding to the soluble IL-6 receptor (sIL-6R), thereby enabling trans-signaling and extending cellular responsiveness to IL-6 [43]. In this context, Sowa-Kućma et al. found that TRD is characterized by elevated sIL-6R levels compared with non-TRD patients [44]. Moreover, studies have shown that IL6R polymorphisms may affect treatment response in MDD patients [17]. Furthermore, a meta-analysis conducted by Liu et al. found that patients with MDD who responded to antidepressant treatment exhibited lower baseline IL-8 levels compared with non-responders. Additionally, antidepressant treatment was associated with a significant reduction in TNF-α levels only in responders, who also demonstrated a significantly greater decrease in TNF-α levels compared with non-responders [45]. Collectively, these biomarkers may predict reduced treatment response to conventional antidepressants, suggesting that the MDD “inflammatory subtype” may overlap with the biological profile observed in TRD patients.

Dysregulation of the HPA axis also plays a key role in the pathophysiology of DDs. The HPA axis is a complex neuroendocrine system that coordinates the physiological response to stress, primarily through cortisol secretion. In response to a stressor, the hypothalamus releases corticotropin-releasing hormone (CRH), which triggers the anterior pituitary gland to secrete adrenocorticotropic hormone (ACTH). ACTH subsequently stimulates the adrenal glands to produce cortisol, which plays a central role in the stress response [46]. Preclinical animal models of depression often involve HPA axis dysregulation, with chronic hyperactivation reflecting key neuroendocrine and behavioral features of DDs. Animal models include chronic corticosterone (CORT) or adrenocorticotropic hormone (ACTH) administration, chronic stress paradigms (CSDS, CUMS, CRS) and genetic models, such as the Wistar Kyoto (WKY) model, all of which lead to persistent HPA axis activation and depressive-like phenotypes [47].

Moreover, neuroinflammation and HPA axis dysregulation are characterized by a complex bidirectional relationship, underpinned by overlapping biological pathways [48]. In this context, Benatti et al. highlighted that the interaction between HPA axis hyperactivity and neuroinflammation is crucial in the etiopathogenesis of MDD, suggesting that targeting these pathways may improve antidepressant efficacy [49]. Chronic stress and consequent HPA axis hyperactivity can increase the production of proinflammatory cytokines, which are known to contribute to mood dysregulation. Conversely, cytokines released during inflammatory responses may act on the hypothalamus, stimulating further CRH, ACTH, and cortisol release and thereby amplifying HPA axis activation. Notably, sustained hypercortisolemia may additionally lead to glucocorticoid receptor (GR) desensitization, impairing negative feedback regulation of the HPA axis [46].

This mechanism appears to be particularly relevant in TRD, where chronic HPA axis hyperactivation, impairment of negative feedback regulation and neuroinflammation may contribute to treatment-resistant symptoms [50]. In this context, Verala et al. used the ACTH-induced depression model in male rats to investigate the relationship between anhedonia-like behavior, chronic low-grade inflammation and bupropion treatment resistance, with doses of 10 mg on day 23 or 20 mg on day 24. They demonstrated that chronic ACTH elicited both an antidepressant resistant and anhedonia-like phenotype, which were strongly associated with increased peripheral IL-6 levels [51]. Moreover, key evidence suggests that the dynamic reactivity of the HPA axis during antidepressant treatment may predict treatment response. A study conducted by Beck et al., evaluating repeated measures of the cortisol awakening response (CAR) at baseline and after 10 days of duloxetine treatment, showed that an unfavorable change in CAR predicted non-remission at 6 weeks. Conversely, greater reductions in CAR levels were associated with more pronounced clinical improvement in DDs [52]. These findings indicate that normalization of HPA axis function during antidepressant treatment may represent a biomarker of treatment responsiveness; however, chronic HPA axis dysregulation and impaired cortisol dynamics may contribute to treatment resistance, supporting the thesis that TRD is characterized by persistent and potentially less reversible dysregulation of the HPA axis.

Moreover, studies indicate the potential therapeutic role of metyrapone, an anti-glucocorticoid agent that inhibits cortisol synthesis. Evidence shows that metyrapone may induce a significant reduction in depressive symptoms in patients with MDD [53]. However, in a double-blind, placebo-controlled trial, metyrapone augmentation of ongoing serotonergic antidepressants did not improve depressive symptoms compared with placebo in patients with TRD [54]. Similarly, Strawbridge et al. demonstrated that metyrapone augmentation was not efficacious in patients with TRD. The study further showed that metyrapone treatment led to increased IL-6 levels, which were associated with poorer depressive outcomes. Metyrapone failed to reduce cortisol concentrations, possibly due to compensatory activation of the glucocorticoid system, with IL-6-mediated mechanisms potentially contributing to the observed lack of clinical efficacy [55]. From this perspective, persistent HPA axis activation in TRD may not solely represent a primary pathogenic driver, but rather a maladaptive persistence of an initially protective stress-response mechanism. In contrast, restoration of dynamic cortisol regulation during effective treatment may serve as a biomarker of neurobiological resilience. This conceptual framework emphasizes that treatment resistance may emerge when adaptive stress-regulatory systems lose flexibility and fail to recalibrate.

### 3.3. Oxidative Stress, Iron Dysregulation, and Ferroptosis

DDs are also significantly influenced by oxidative stress in their pathophysiology. Oxidative stress results from an imbalance between the overproduction of ROS (e.g., superoxide anions, hydrogen peroxide (H_2_O_2_), and hydroxyl radicals) and insufficient antioxidant defense mechanisms [56]. Moreover, a growing body of evidence indicates a bidirectional relationship between neuroinflammation and oxidative stress. Excessive ROS generation and concomitant depletion of antioxidant systems promote microglial activation via the nuclear factor kappa-light-chain-enhancer of activated B cells (NF-κB) and the NLRP3 inflammasome signaling pathways, leading to elevated levels of proinflammatory cytokines (e.g., IL-1β and TNF-α) and sustained neuroinflammatory responses. In turn, neuroinflammation may activate microglia, upregulating NADPH oxidase 2 (NOX2), increasing ROS production and lipid peroxidation, thereby further intensifying oxidative stress [57]. Additionally, elevated ROS levels modulate HPA axis feedback by desensitizing glucocorticoid receptors, leading to HPA axis hyperactivity and impaired regulatory control. Conversely, HPA axis hyperactivity further promotes ROS production, resulting in a self-perpetuating vicious cycle of oxidative stress and neuroendocrine dysregulation [58].

Studies have also shown a positive correlation between oxidative stress severity and depressive symptoms, as well as a negative correlation between antioxidant status and depression risk [14]. Moreover, in MDD, increased levels of oxidative markers, such as lipid peroxidation products (LPO), TBARS, 8-isoprostane, malondialdehyde (MDA), F2-isoprostanes (F2-IsoP), and 8-hydroxy-2′-deoxyguanosine (8-OHdG), are observed, along with reduced activity of antioxidant enzymatic systems, including glutathione (GSH), catalase (CAT), superoxide dismutase (SOD), and glutathione peroxidase (GPX), as well as decreased expression of nuclear factor erythroid 2-related factor 2 (Nrf2), a key regulator of cellular antioxidant defenses [59,60,61]. Emerging evidence indicates that more severe MDD correlates with higher 8-OHdG levels compared with milder forms, suggesting that oxidative stress may serve as a potential biomarker of episode persistence and disease severity [62].

Given the above, Santos et al. found that CORT-induced depression resulted in increased H_2_O_2_ levels and decreased catalase activity in the HP, along with elevated serum levels of IL-17 and IFN-γ. Moreover, these mice did not respond to fluoxetine treatment (10 mg/kg/day). These findings suggest that chronic CORT administration, which reflects the effects of elevated glucocorticoids, promotes hippocampal oxidative stress and neuronflammation, processes that are all closely linked and may synergistically contribute to antidepressant treatment resistance [63]. In line with these findings, Lindqvist et al. reported that patients who did not respond to SSRI treatment showed a significant increase in 8-OHdG over the course of treatment [59]. Moreover, non-responders to SSRI treatment had higher F2-IsoP levels than responders, both at baseline and after eight weeks of treatment [60]. Rybka et al. found that increased H_2_O_2_ levels were characteristic of patients with recurrent, severe DDs [64]. Additionally, it was shown that H_2_O_2_ levels are elevated in patients with TRD, possibly due to abnormal estrogen metabolism contributing to oxidative stress in this group of patients [65]. Consistently, Sowa-Kućma et al. demonstrated increased TBARS levels in individuals with TRD compared with those with MDD, further suggesting a more pronounced oxidative imbalance in TRD [44]. Emerging evidence suggests that patients with TRD exhibit dysregulated iron homeostasis in the CNS, including alterations in striatal tissue iron concentration [66]. Dysregulated iron metabolism promotes the generation of iron-dependent reactive species, which contribute to oxidative stress and lipid peroxidation—processes that have also been observed in TRD [44]. The resulting oxidative stress may induce ferroptosis, an iron-dependent form of regulated cell death characterized by lipid peroxidation, thus compromising antioxidant defense systems, including Nrf2-mediated pathways [67]. In this context, post-mortem studies of suicide victims (representing the most severe end of the TRD spectrum) have shown alterations in Nrf2 protein levels, suggesting a critical role of impaired antioxidant regulation in the severity and persistence of TRD [68]. Since TRD patients typically exhibit high levels of oxidative stress and reduced responsiveness to conventional treatment, oxidative stress may represent a mechanism contributing to the perpetuation of treatment resistance. Furthermore, while compensatory antioxidant mechanisms appear generally preserved in MDD, TRD appears to be characterized by persistent, maladaptive oxidative stress pathways. It is therefore plausible that oxidative stress markers observed in early or moderate stages of MDD may reflect an active compensatory upregulation of redox-regulatory systems. Only when these mechanisms become chronically insufficient or dysregulate, as may occur in TRD, does oxidative stress transition from an adaptive signal to a maladaptive driver of cellular dysfunction.

### 3.4. Tryptophan–Kynurenine Pathway and Glutamatergic Dysregulation

Neuroinflammation may contribute to metabolic dysregulation and altered neurotransmitter homeostasis. Inflammatory mediators, including cytokines such as interferon-γ (IFN-γ), activate the enzyme indoleamine 2,3-dioxygenase (IDO-1). This shifts tryptophan (TRP) metabolism toward the kynurenine (KYN) pathway, increasing the production of downstream metabolites, including the neurotoxic compound quinolinic acid (QA) [69,70]. This enzymatic activation is primarily driven by pro-inflammatory signaling and represents a key mechanistic link between immune activation and altered neurotransmission.

The accumulation of neuroactive kynurenine metabolites may contribute to mood disorders and potentially to treatment resistance. In one study, rats subjected to CUMS and subsequently treated with fluoxetine (10 mg/kg/day, intraperitoneally) were identified as non-responders (this subgroup was designated as a fluoxetine-resistant depression–FRD model). It should be noted that although animal models cannot fully replicate the clinical complexity of TRD, they allow investigation of selected mechanisms underlying non-response to antidepressants [29] The FRD group was subsequently treated with Kai Xin San (KXS) (491 mg/kg/day, orally) for two weeks. KXS is a traditional herbal formula with reported antidepressant properties, which in preclinical studies has demonstrated potential therapeutic effects in models of treatment resistance, possibly through modulation of tryptophan metabolism [71]. KXS alleviated depressive-like behavior in FRD rats and partially restored tryptophan–kynurenine metabolic balance, both peripherally and in the hippocampus, by reducing the expression/activity of IDO-1, tryptophan 2,3-dioxygenase (TDO-2), and kynurenine-3-monooxygenase (KMO). This was accompanied by normalization of metabolite ratios, including KYN/TRP and KYNA/QA, as well as decreased QA levels. Changes in 5-HT concentrations were also observed, suggesting partial restoration of serotonergic homeostasis [72]. These findings indicate that modulation of abnormal TRP metabolites may be relevant not only for restoring serotonergic balance but also for regulating glutamatergic transmission via the kynurenic acid (KYNA)/quinolinic acid (QA) axis, which is increasingly recognized as an important pathway in the pathophysiology of TRD [73].

In this context, two opposing metabolites play a central role: KYNA, an endogenous NMDAR antagonist, and QA, an NMDAR agonist. Importantly, QA’s neurotoxic effects are not limited to direct NMDAR activation; QA may also impair astrocytic function, including inhibition of glutamate uptake from the synaptic cleft. This can lead to extracellular glutamate accumulation [74]. The imbalance between KYNA and QA under conditions of neuroinflammation and oxidative stress may contribute to NMDAR dysregulation, excitotoxicity, impaired synaptic plasticity, and sustained inflammatory signaling. These mechanisms are considered potential contributors to persistent depressive symptoms and TRD. Additionally, genetic variation in glutamatergic signaling may influence treatment response. Variants in the *GRIN2B* gene (encoding the GluN2B NMDAR subunit) have been associated with antidepressant response and may represent a prognostic factor in MDD and TRD [16].

Glutamate exerts its effects primarily through ionotropic receptors, including not only NMDAR but also AMPAR. The balanced interaction between these receptor systems is essential for long-term potentiation (LTP), synaptic remodeling, and the regulation of neurotrophic factors, particularly brain-derived neurotrophic factor (BDNF) [75]. Accordingly, modulation of NMDAR activity is not only mechanistically relevant to the pathophysiology of TRD but also represents a validated and clinically established therapeutic strategy. Robust evidence supporting this concept comes from studies of ketamine, a non-competitive NMDAR antagonist, which has demonstrated rapid antidepressant effects in patients with TRD [76].

In a randomized, double-blind, active-controlled crossover study conducted by Glue et al., intramuscular racemic ketamine (0.5 mg/kg and 1 mg/kg) was compared with fentanyl (50 μg) in 25 patients with TRD. Each participant received three interventions at one-week intervals. Both ketamine doses resulted in significantly greater reductions in HADS-Depression and HADS-Anxiety scores compared with fentanyl, while maintaining an acceptable safety profile [77]. These findings support the clinical efficacy of glutamatergic modulation in TRD and underscore the importance of further investigating the underlying neurobiological mechanisms of treatment resistance.

Impaired neuroplasticity is increasingly recognized as a core feature of TRD. Growing evidence indicates that neuroinflammation, HPA axis dysregulation, oxidative stress, and glutamatergic imbalance collectively contribute to structural and functional brain alterations. These interconnected processes converge particularly in the hippocampus, a stress-sensitive brain region essential for adult neurogenesis and BDNF-dependent synaptic plasticity [13,78]. BDNF plays a central role in neuronal survival, differentiation, synaptic function, and neurogenesis, and dysregulation of BDNF signaling is consistently associated with DDs [78]. For example, Wistar Kyoto (WKY) rats exposed to CMS develop a TRD-like phenotype characterized by behavioral deficits, reduced neurogenesis, and decreased BDNF expression [79].

**Figure 2:**
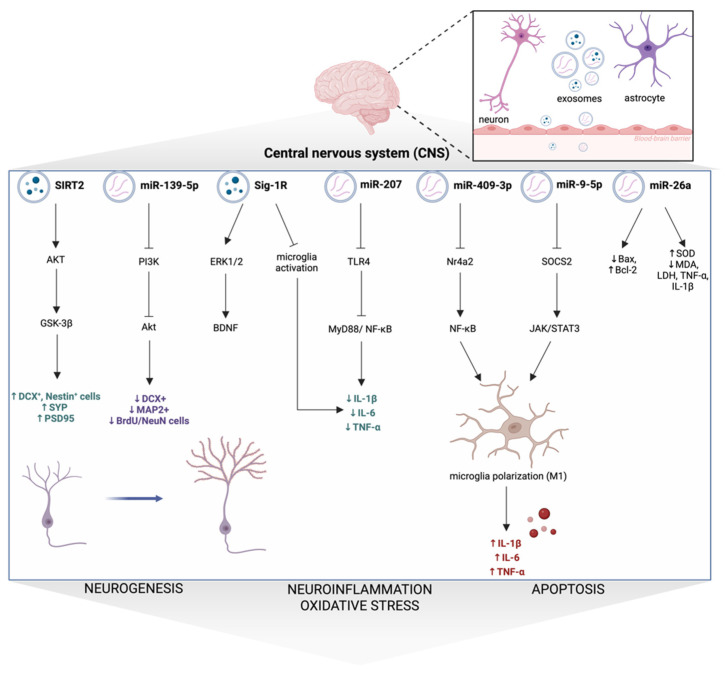
Exosomes in the pathophysiology of TRD. SIRT2 activates the AKT/GSK-3β pathway, promoting neurogenesis as evidenced by increased expression of DCX^+^, Nestin^+^ cells, SYP, and PSD95. miR-139-5p inhibits the PI3K/Akt pathway, leading to reduced neuronal differentiation markers (DCX^+^, MAP2^+^, BrdU/NeuN^+^ cells). Sig-1R activates ERK1/2 signaling, enhances BDNF expression, and suppresses microglial activation, thereby reducing pro-inflammatory cytokines (IL-1β, IL-6, TNF-α). miR-207 modulates TLR4/MyD88/NF-κB signaling, thereby contributing to decreased neuroinflammation. miR-409-3p regulates Nr4a2-dependent NF-κB activation, while miR-9-5p influences SOCS2 and the JAK/STAT3 pathway, affecting microglial polarization toward pro-inflammatory M1 phenotype. miR-26a exhibits anti-apoptotic and antioxidant effects by increasing SOD levels and reducing Bax, MDA, LDH, TNF-α, and IL-1β, while upregulating Bcl-2. Collectively, these pathways highlight the interplay between neurogenesis, neuroinflammation, oxidative stress, and apoptosis, which all may underlie TRD pathophysiology. Abbreviations: AKT-Protein kinase B; BDNF-Brain-Derived Neurotrophic Factor; Bax-Bcl-2-Associated X protein; Bcl-2-B-cell lymphoma 2; BrdU-5-Bromo-2′-deoxyuridine; DCX-Doublecortin; ERK1/2-Extracellular Signal-Regulated Kinases 1 and 2; GSK-3β-Glycogen Synthase Kinase 3 beta; IL-1β-Interleukin 1 beta; IL-6-Interleukin 6; JAK-Janus Kinase; LDH-Lactate Dehydrogenase; MAP2-Microtubule-Associated Protein 2; MDA-Malondialdehyde; miR-microRNA; MyD88-Myeloid Differentiation Primary Response 88; Nestin-Neural stem cell marker Nestin; NeuN-Neuronal Nuclei (Neuronal Nuclear Antigen); NF-κB-Nuclear Factor kappa-light-chain-enhancer of activated B cells; Nr4a2-Nuclear Receptor Subfamily 4 Group A Member 2; PI3K-Phosphoinositide 3-Kinase; PSD95-Postsynaptic Density Protein 95; Sig-1R-Sigma-1 Receptor; SOCS2-Suppressor of Cytokine Signaling 2; SIRT2-Sirtuin 2; SOD-Superoxide Dismutase; STAT3-Signal Transducer and Activator of Transcription 3; SYP-Synaptophysin; TLR4-Toll-Like Receptor 4; TNF-α-Tumor Necrosis Factor alpha. (→)—activated; (⊣)—inhibited; (↑)—increase; (↓)—decrease.

From a clinical perspective, lower baseline BDNF levels have been associated with a poorer response to selective serotonin reuptake inhibitors (SSRIs), while the BDNF Val66Met polymorphism has been linked to differential treatment outcomes, with the Val/Val genotype showing more favorable responses than Val/Met carriers [80]. Neuroimaging studies further demonstrate reduced hippocampal volume in individuals with TRD compared with healthy controls [18]. In addition, structural imaging analyses have reported gray matter volume reductions in frontal, temporal, limbic, and cingulate regions in TRD, together with altered large-scale network connectivity. Of particular relevance is reduced connectivity within the default mode network (DMN), including temporal and parietal regions, observed in TRD compared with treatment-responsive depression [19]. Collectively, these findings indicate that TRD is associated with convergent molecular, structural, and functional brain abnormalities that may contribute to persistent symptoms and diminished treatment responsiveness (Figure 2).

## 4. Exosome-Based Assessment in DDs

### 4.1. Biological Sources of Exosomes in DDs

Exosome-based approaches have emerged as a promising research direction for understanding the molecular basis of various diseases (including DDs), offering a minimally invasive window into both central and peripheral pathophysiological processes. However, variability in biological sources and analytical methodologies, alongside the lack of standardized protocols, remains a major barrier to clinical application. Generally, EXOs for analytical purposes are derived from two principal sources: biological fluids and conditioned cell culture media (in vitro systems). Among biological fluids, serum, platelet-rich plasma, cerebrospinal fluid, saliva, urine, and breast milk are most frequently utilized for EXO isolation; however, the yield is influenced by both the cellular origin and the experimental design, including preanalytical handling and preconditioning conditions applied to the cells or fluids.

Blood is the most widely used biological source in EXOs research. However, their isolation requires rigorous preanalytical handling, as both serum and plasma are highly susceptible to contamination. In addition, platelet activation, hemolysis, and coagulation-associated processes may lead to the formation of additional vesicles or the sequestration of native EXOs, thereby modifying the final vesicular profile. Moreover, the substantial abundance of lipoproteins, extracellular vesicles, and proteins in blood significantly complicates the attainment of highly purified exosome preparations, even after repeated centrifugation and filtration steps [81].

Cerebrospinal fluid (CSF) is a particularly valuable biofluid for the isolation and analysis of neuron-derived exosomes and other CNS-associated extracellular vesicles, due to its close proximity to the brain parenchyma and relatively low cellular and protein content. CSF contains approximately 5 cells per microliter and has a protein concentration roughly 200-fold lower than that of plasma, reducing background interference and facilitating downstream molecular analyses. Nevertheless, even minimal blood contamination during lumbar puncture can substantially alter the vesicular and protein composition of CSF, introducing plasma-derived proteins and extracellular vesicles that may confound exosome isolation and biomarker quantification. In addition, the limited sample volume, together with the invasive nature of the procedure, restricts repeated sampling and longitudinal monitoring, and may necessitate sample pooling, thereby reducing the ability to assess inter-individual variability [82].

In this context, the isolation of exosomes from saliva or urine represents a minimally invasive alternative to approaches based on blood or cerebrospinal fluid. Such strategies may be particularly valuable in specific patient populations, including women with peripartum depression (approximately 5% develop TRD) as well as older adults with MDD, in whom up to half of patients may progress to TRD [83,84]. Nevertheless, isolation protocols for EXOs derived from saliva and urine remain less standardized than those established for blood- or CSF-based approaches, contributing to methodological heterogeneity and reduced reproducibility across studies. Importantly, accumulating evidence suggests that EXOs originating from different biological sources may differ in their molecular composition and biophysical properties. In line with this, Mohammadinasr et al. demonstrated that CSF- and serum-derived EXOs display distinct molecular cargo, reflected in divergent miRNA expression profiles in patients with relapsing-remitting multiple sclerosis [85].

### 4.2. Isolation Methods and Technological Advances in EXOs Research

The diagnostic utility of EXOs is critically dependent on the methods employed for their isolation, characterization, and detection. Traditional isolation strategies in preclinical and clinical research include ultracentrifugation and immunoaffinity- and precipitation-based extraction kits; additional widely used approaches include ultrafiltration and size exclusion chromatography. While conventional exosome extraction techniques remain common, they are often constrained by low isolation yield, limited purity, and prolonged processing times [86].

Recent technological innovations have provided a diverse array of EXO isolation strategies that improve purity, yield, and the ability to capture specific vesicle subpopulations [81,86,87]. Microfluidic platforms enable size-based separation using acoustic nanofilter systems (label-free microfluidic platforms) or selective capture through integrated antigen–antibody reactions and magnetic forces on microfluidic chips (immunoaffinity-based microfluidics) [86,87]. Nanolithography is based on engineered nanoscale surface architectures, such as lipid or functionalized patterns, to enable highly selective EXOs capture through mechanisms including membrane interactions or affinity-based binding [86]. In contrast, electro-deposition relies on electrochemical properties and localized electric fields to drive controlled accumulation and enrichment of vesicles on conductive surfaces [87]. Immunomagnetic beads utilize antibody-coated magnetic particles for targeted isolation of defined exosome subsets, while covalent chemistry allows stable attachment of vesicles via surface-reactive groups [81,86].

In addition to these approaches, asymmetric flow field-flow fractionation (AF4) separates EXOs in a continuous, label-free manner based on size, density, and Brownian motion, enabling high-resolution fractionation of extracellular vesicles [88]. Unlike conventional bulk isolation techniques, AF4 functions primarily as a high-resolution separation and characterization platform, allowing discrimination of distinct vesicle populations with improved analytical precision [88,89]. This method enabled the separation of EXO subpopulations, distinguishing large exosome vesicles (Exo-L, 90–120 nm), small exosome vesicles (Exo-S, 60–80 nm), and a population of non-membranous nanoparticles termed “exomers” (~35 nm) [89]. These nanoparticle subsets exhibit distinct molecular and functional characteristics, including differences in protein, lipid, metabolite, and nucleic acid composition, as well as divergent organ biodistribution patterns [89]. Exo-S likely represents canonical exosomes of endosomal origin, whereas Exo-L may include non-canonical exosomes or vesicles derived from alternative subcellular pathways, such as plasma membrane budding [88,89]. Additionally, exomers appear to lack a surrounding lipid bilayer, suggesting a distinct biogenetic mechanism and cargo packaging pathway compared with membrane-bound exosomes [89]. Compared with EXOs subpopulations, exomers were enriched in lipids such as triglycerides and ceramides and showed associations with metabolic pathways including glycolysis and mTOR signaling [88,90]. In contrast, Exo-S and Exo-L contained proteins related to endosomal function and secretory pathways, as well as components linked to cytoskeletal organization (e.g., mitotic spindle regulation) and IL-2/STAT5 signaling, respectively [88]. These findings underscore the substantial heterogeneity of extracellular vesicle populations and highlight the need for standardized methodological frameworks for their classification and analysis [89].

A deeper understanding of EXOs subpopulations may facilitate the identification of specific molecular markers, thereby supporting more precise isolation and functional characterization in translational research [81,89]. In the context of TRD and broader psychiatric disorders, AF4 may aid in identifying EXO subtypes potentially associated with neuroinflammatory, metabolic, or synaptic dysregulation [34,88]. Such insights could contribute to the development of novel biomarkers for disease stratification and treatment response and may inform the design of targeted EV-based therapeutic strategies [91,92]. However, further studies are required to determine the prevalence and functional relevance of specific EXOs subpopulations in DDs, particularly TRD [93]. To date, AF4 has not been systematically applied in studies investigating EXOs subpopulations in depression.

Collectively, these approaches offer complementary advantages: AF4 and label-free microfluidic systems enable gentle, unbiased separation, whereas immunoaffinity-, nanostructure-, and covalent-based strategies facilitate selective enrichment of rare vesicle subsets, supporting their potential utility in biomarker discovery and functional studies [81,87,94].

### 4.3. Characterization and Detection of Exosomes

Conventional approaches for exosome detection include enzyme-linked immunosorbent assay (ELISA), mass spectrometry and polymerase chain reaction (PCR), while emerging quantitative techniques are based on microfluidic chips, gene chips and nanofluorescent probes [87]. These methods differ in sensitivity, specificity, and suitability for different analytical applications, ranging from protein quantification to nucleic acid profiling.

EXOs are commonly characterized by the expression of canonical markers, including tetraspanins (CD9, CD63, CD81) and endosomal proteins such as HSP70, HSP90, TSG101, and Alix [81,89]. According to current consensus guidelines (MISEV), these markers should be interpreted as enrichment indicators rather than as exclusive or definitive markers of exosomes. Additionally, neuron-derived extracellular vesicles (NDEVs) may be enriched from peripheral blood using neuronal markers, e.g., L1 cell adhesion molecule (L1CAM), β-III-tubulin, and VAMP2 [95,96]. However, these isolation strategies require careful validation. The use of L1CAM as a single selection marker for NDEVs remains controversial, as most L1CAM detected in human plasma or cerebrospinal fluid exists in a soluble form rather than being vesicle-associated. Moreover, L1CAM is expressed not only by neurons but also by peripheral cell types such as Schwann cells and renal epithelial cells, which may result in contamination of NDEV preparations with non-neuronal EVs, underscoring the need for improved standardization and multi-marker validation approaches [97].

Conversely, Nogueras-Ortiz et al., using Simoa assays and microscopy, showed that L1CAM co-localizes with both EV markers (tetraspanins) and neuronal markers (β-III-tubulin, VAMP2 and GAP43). In human plasma, 3.8% of all EVs were L1CAM-positive; among these, 67.4% carried tetraspanins (confirming their EV identity), 33.6% expressed VAMP2 and 54.0% expressed β-III-tubulin. Importantly, soluble L1CAM fragments in plasma do not bind to the surface of EVs, ruling out non-specific contamination. A large proportion of immunocaptured L1CAM-positive EVs co-expressed tetraspanins and VAMP2, while being negative for ASGR2, a hepatocyte membrane marker and a major source of peripheral L1CAM, confirming that most isolated EVs are of neuronal origin. Nevertheless, approximately one-third of L1CAM-positive EVs may still derive from non-neuronal cells, indicating the need to further improve isolation methods, for example, by including additional neuronal markers [96].

### 4.4. Biogenesis and Heterogeneity of Small Extracellular Vesicles (sEVs)

Small extracellular vesicles are generally defined as vesicles with a diameter below 200 nm, although size alone is not sufficient to define their biological origin or subtype. In the literature, the terms “sEVs” and “EXOs” are frequently used interchangeably (for the sake of simplicity and consistency, we aim to use the unified term “EXOs” throughout this review); however, these terms are not synonymous and should not be considered equivalent. The sEV fraction comprises both exosomes and small ectosomes. The fundamental distinction between these vesicle types lies in their mechanisms of biogenesis. Exosomes originate from the endosomal pathway and are released following the fusion of multivesicular bodies (MVBs) with the plasma membrane. In contrast, ectosomes (also referred to as microvesicles or microparticles) are generated independently of MVBs through direct outward budding and shedding from the plasma membrane. Because exosomes and ectosomes arise from distinct subcellular compartments, they may carry different molecular cargo and therefore exert partially distinct biological functions [89].

This distinction is particularly relevant in studies of vesicle-based therapies derived from stem cells, where exosomes have, in some experimental models, demonstrated more pronounced therapeutic effects compared with ectosomes [98,99]. However, this observation remains context-dependent and has not been conclusively established across all models and disease settings. A major limitation is the technical difficulty in separating exosomes from ectosomes of overlapping size ranges in biofluids or conditioned media. Furthermore, there are currently no universally accepted protein markers that unequivocally distinguish exosomes from ectosomes, and no experimental tools provide complete specificity for either biogenetic pathway.

A recent study by Mathieu et al. identified candidate markers that may assist in discriminating sEV subtypes [100]. For example, EVs containing only CD9 or CD81 but lacking CD63 are more likely to represent non-endosomal vesicles (i.e., ectosomes), whereas vesicles expressing CD63 in combination with one or more additional tetraspanins may correspond to endosome-derived exosomes. Additionally, proteomic analyses identified LAMP2, PLD3, PLP2, and TSPAN4 as potential exosome-associated markers, whereas L1CAM, DAG1, CD99, SLC3A2, BSG, and DSG3 were proposed as candidate ectosome-associated markers [100]. However, the cell-type specificity, reproducibility, and broader applicability of these markers require further validation in independent studies. Importantly, affinity-based protocols targeting the tetraspanins CD9, CD63, and CD81 do not selectively isolate exosomes, as antibody-based capture enriches partially overlapping vesicle populations with heterogeneous molecular compositions. Moreover, not all EVs express tetraspanins; therefore, tetraspanin-based enrichment does not represent the entire EV population.

Neurons and microglial cells can produce both exosomes and ectosomes. The subcellular origin of these vesicles may influence whether their cargo is enriched in inflammatory mediators, regulatory miRNAs, synaptic proteins, or other functional molecules [101]. Accordingly, the development of standardized isolation protocols and the identification of well-validated, subtype-associated biomarkers are essential for the accurate characterization of sEV cargo and for clarifying their role in the pathophysiology of TRD.

### 4.5. Standardization Challenges and Clinical Translation

Taken together, exosome-based approaches represent a promising, minimally invasive strategy for investigating the central and peripheral pathophysiological mechanisms underlying DDs. However, their potential translational and clinical utility remains constrained by heterogeneity in biological sources, methodological variability in isolation and detection techniques, and the absence of universally standardized protocols. Consequently, the reliability and comparability of exosome-derived biomarkers across studies are still limited. To date, no systematic investigations have specifically evaluated exosome isolation from saliva or urine in patients with DDs, and comparative studies assessing the biophysical and molecular characteristics of sEVs derived from different biological fluids (e.g., blood, cerebrospinal fluid, saliva, and urine) in DDs are lacking.

Importantly, the development and implementation of rigorously standardized and validated methodologies are essential for reliable discrimination of sEV subpopulations, particularly given their demonstrated biogenetic and molecular heterogeneity.

## 5. Preclinical Insights into the Therapeutic Potential of Exosome-Derived Interventions in Depressive Disorders

EXOs production takes place both in vivo in biological fluids and in vitro in cell culture medium. Cellular sources include stem cells derived from bone marrow, adipose tissue and umbilical cord blood, alongside neural cells (e.g., oligodendrocytes and neural stem cells). EXOs derived from these cells exert therapeutic effects and may play a key role in DDs by attenuating depressive-like behaviors in preclinical models (see Table 1). As previously mentioned, alterations in neuroplasticity play a key role in the pathophysiology of DDs. Nevertheless, the problem remains complex, as neuroplasticity involves multiple molecular pathways that may be dysregulated or impaired. Neuroplasticity can be broadly classified into mechanisms acting at the cellular and molecular levels. At the cellular level, neuroplasticity occurs at the subcellular levels of neurogenesis, synaptogenesis, and dendritogenesis [102]. New neurons are continuously generated in two brain regions: the subgranular zone of the hippocampal dentate gyrus and the subventricular zone of the lateral ventricles. They arise from neural progenitor cells and progress through distinct morphological and functional stages prior to maturation [102]. Glial fibrillary acidic protein (GFAP), nestin, doublecortin (DCX) and neuronal nuclei (NeuN) serve as key molecular markers for identifying and phenotyping newborn cell populations in the adult dentate gyrus. GFAP is commonly used as an astrocyte marker, as it constitutes the major component of the astrocytic cytoskeleton and the scaffolding. Nestin marks proliferating progenitor cells at early developmental stages, whereas DCX labels postmitotic neuroblasts and immature neurons. NeuN serves to identify fully differentiated, mature neurons. Collectively, these markers provide a framework for monitoring neurogenesis [103]. It is well established that DDs are associated with a marked reduction in the proliferation of neural stem cells (NSCs), neural progenitor cells, and neuroblasts, which is reflected by decreased numbers of DCX+ and Nestin+, NeuN+ cells [104]. Moreover, Xie et al. reported that patients with TRD exhibited significantly lower levels of DCX in neuron-derived extracellular vesicles (NDEVs) compared to healthy controls. Interestingly, exploratory analyses demonstrated that reduced DCX levels were correlated with more severe depressive symptoms and baseline cognitive deficits, suggesting a link between diminished neurogenesis and TRD [105]. Synaptophysin (SYP/SYN), growth-associated protein-43 (GAP-43), and post-synaptic density protein-95 (PSD95) are key synaptic proteins. While SYP and GAP-43 are localized at the presynaptic terminal, PSD95 is a part of a protein complex located in the postsynaptic membrane. In DDs, PSD95, SYP, and GAP-43 levels are consistently reduced in the HP and PFC, reflecting impaired synaptogenesis and neuroplasticity [106]. Importantly, severe stress-induced depressive-like behaviors in rats were associated with decreased PSD-95 expression in the PFC and increased PSD-95 in the AMY, highlighting region-specific synaptic alterations in TRD. Ketamine (single dose; 10 mg/kg, i.p.), but not fluoxetine (28 days; 10 mg/kg, i.p.), restored these changes, suggesting a mechanistic link between PSD-95 dysregulation and TRD-like phenotypes [107]. Furthermore, upregulation of Myd88 in medial PFC has been shown to impair dendritic growth and reduce spine density, which was associated with depressive-like behaviors [108]. At the molecular level, neuroplasticity is mediated by highly coordinated intracellular signaling pathways that regulate neuronal survival, synaptic remodeling and metabolic adaptation. The phosphatidylinositol-3-kinase (PI3K)/AKT pathway is a key regulator of neurogenesis, neuronal survival and neural stem cell fate. Reduced PI3K and AKT expression, as well as decreased AKT phosphorylation, have been observed in DDs, leading to impaired viability and outgrowth of newborn neurons in the dentate gyrus. Conversely, enhanced AKT phosphorylation promotes hippocampal neurogenesis. Glycogen synthase kinase-3 beta (GSK-3β), a downstream effector of AKT, is essential in neurogenic regulation. Stress-induced AKT inactivation, observed in DDs, leads to reduced inhibitory phosphorylation of GSK-3β at Ser9, thereby diminishing cytoplasmic accumulation and stabilization of β-catenin and contributing to neurogenesis deficits [90]. Peroxisome proliferator-activated receptor gamma co-activator (PGC-1α) is a key transcriptional regulator of mitochondrial biogenesis that exerts its effects, in part, through nuclear respiratory factor 1/2 (NRF1/2) upregulation and the subsequent activation of mitochondrial transcription factor (TFAM). Activation of PGC-1α promoter has been shown to increase BDNF expression in HP, likely via extracellular signal-regulated kinases (ERKs)- and cAMP response element-binding protein (CREB)-mediated signaling. In turn, BDNF-dependent activation of PGC-1α, NRF1/2 and TFAM promotes mitochondrial biogenesis and supports synapse formation in hippocampal neurons [109]. Additionally, the PGC-1α/NRF1/TFAM signaling pathway may also be activated via increased phosphorylation of AMP-activated protein kinase (p-AMPK), leading to mitochondrial DNA replication, protein synthesis and mitochondrial biogenesis [110]. Notably, recent studies have highlighted that deficits in neuroplasticity play a key role in DDs, especially TRD. Consistent with this, Wistar Kyoto (WKY) rats subjected to CMS develop a TRD-like phenotype, exhibiting behavioral deficits alongside impaired neurogenesis, decreased BDNF levels, suppressed PI3K/AKT signaling and dysregulated MAPK/ERK activity, functionally reflecting key molecular and behavioral features of TRD, providing a basis for future therapeutic strategies [79]. Collectively, growing evidence indicates that exosome-derived interventions promote neuroplasticity, thereby alleviating depressive-like symptoms (see Table 1).

Moreover, exosome-derived interventions may exert antidepressant effects by attenuating neuroinflammation and oxidative stress through reducing pro-inflammatory cytokine levels (e.g., IL-1β, IL-6, TNF-α), decreasing inducible nitric oxide synthase (iNOS), Iba-1, MDA, LDH, NO levels and NF-κB activity and increasing SOD and anti-inflammatory cytokine (IL-10) levels (see Table 1). Autophagy is a fundamental catabolic multistep process in which intracellular proteins and other cytoplasmic components are sequestered into autophagosomes and subsequently delivered to lysosomes for degradation. This cellular degradation and recycling pathway is critical for neuronal homeostasis, and its dysregulation is increasingly recognized as a contributing mechanism in DDs through impaired neuroplasticity and enhanced neuroinflammatory signaling. AMPK and mammalian target of rapamycin kinase (mTOR) function as central signaling pathways controlling autophagy by regulating autophagy-related proteins, such as microtubule-associated protein light chain 3 (LC3B) and Beclin1. Increased mTOR signaling has been associated with attenuation of autophagy-related markers (e.g., LC3BII/I, Beclin1). Conversely, AMPK activation via phosphorylation of its α-subunits at Thr-172 may inhibit mTOR activity, thereby enhancing autophagy [118]. In DDS, autophagy dysregulation manifests as either suppressed initiation due to mTOR hyperactivity or aberrant activation of the AMPK-mTOR signaling pathway, with both states contributing to impaired synaptic plasticity and neuroinflammation [118]. Notably, growing evidence suggests that antidepressant treatment may mitigate dysregulation of autophagy. Both esketamine and ketamine may activate mTOR, thereby modulating autophagy and promoting synaptic plasticity, which contributes to their rapid antidepressant effect [119,120]. Moreover, Johnston et al. identified that in iPSC-derived neurons from TRD patients, reelin specifically upregulates mTORC1 [121]. Furthermore, Singh et al. highlighted that peripheral immune cell mTOR expression may serve as a biomarker of ketamine response in TRD patients, with ketamine-induced mTOR upregulation correlating with improvements in anhedonia [122]. Apoptosis is a programmed cell death process regulated by Bcl-2 family proteins and caspases that mediates the removal of damaged or dysfunctional cells. Autophagy and apoptosis are closely interconnected processes. Dysregulated autophagy may trigger apoptotic pathways, implicating both in stress-related neuronal dysfunction. Increased neural apoptosis and altered expression of apoptosis-related (Bax, Bcl-2) and signaling (JNK, p38) proteins are observed in DDs [123]. However, exosome-derived interventions may exert an antidepressant effect by normalizing alterations in autophagy signaling (see Table 1).

All original studies included in Table 1 included exosomes characterized according to the MISEV criteria, including analysis of particle size distribution by NTA/DLS, assessment of vesicle morphology by TEM, and identification of protein markers specific to this category (see Table 1).

## 6. Clinical Applications of EXOs in Treatment-Resistant Depression and Personalized Psychiatry

The consistently increasing number of patients who fail to respond to standard antidepressant treatments makes TRD a growing global challenge. Moreover, growing evidence indicates that more than 25% of individuals diagnosed with MDD may develop TRD, with the median time from symptom onset to the recognition of treatment resistance estimated at up to 12 months [124,125]. Furthermore, up to 80% of patients with TRD experience a relapse within a year of remission [124]. Notably, patients with TRD have significantly higher mortality rates due to suicide and accidental overdose compared with individuals with non-resistant MDD, highlighting the urgent need to identify alternative diagnostic and therapeutic strategies [126].

### 6.1. EXOs as Biomarkers

**Figure 3:**
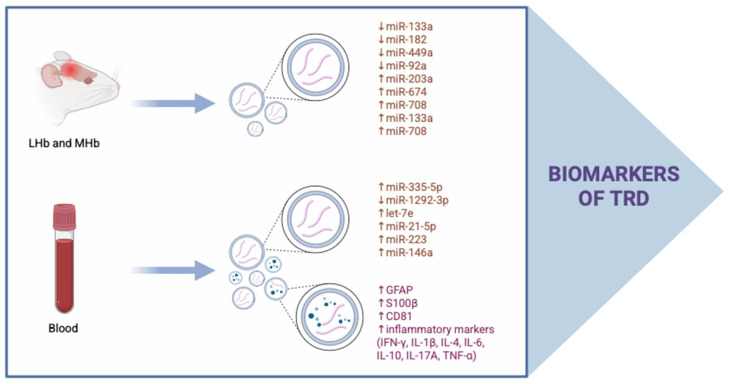
Potential central and peripheral biomarkers in TRD. Analysis of the lateral and medial hypothalamus (LHb, MHb) of mice subjected to a TRD-like phenotype procedure (Wistar Kyoto) reveals downregulation of specific microRNAs, including miR-133a, miR-182, miR-449a and miR-92a, while in the same areas there is an increase in the expression of miR-203a, miR-674, miR-708, miR-133a and miR-708, indicating altered gene regulation promoting drug resistance. In contrast, peripheral blood analysis shows upregulation of circulating miRNAs such as miR-335-5p, miR-21-5p, miR-223, miR-146a and let-7e. This peripheral profile is also characterized by elevated levels of glial damage markers (GFAP, S100β) and the exosomal marker CD81. Furthermore, systemic pathophysiology is confirmed by increased concentrations of pro-inflammatory cytokines, including IFN-gamma, IL-1β, IL-6, IL-10, IL-17A, and TNF-α. Collectively, these distinct molecular signatures of central and peripheral origin highlight the interplay between miRNA dysregulation, glial dysfunction, and inflammation in TRD, providing a comprehensive panel of candidate biomarkers for this phenotype of depression. Abbreviations: LHb—lateral habenula; MHb—medial habenula; miR—microRNA; let-7e—lethal-7e; GFAP—Glial Fibrillary Acidic Protein; S100β—calcium-binding protein; INF—interferon; IL—interleukin; TNF-α—Tumor Necrosis Factor alpha; TRD—treatment-resistant depression. (↑)—increase; (↓)—decrease.

To facilitate the diagnostic process of TRD, EXOs may serve as potential disease biomarkers (Figure 3).

Li et al. demonstrated that two microRNAs in plasma-derived EXOs showed significant differences in expression between patients with TRD and healthy controls. Specifically, the expression of has-miR-335-5p was markedly upregulated, whereas has-miR-1292-3p was substantially downregulated. Notably, target gene GO (gene ontology) enrichment analysis and KEGG (Kyoto Encyclopedia of Genes and Genomes) pathway enrichment analysis revealed that these dysregulated miRNAs are involved in postsynaptic density and axonogenesis, along with axon guidance and cell growth signaling pathways, consistent with previous studies emphasizing the critical role of neuroplasticity in TRD pathogenesis [25]. Additionally, patients with TRD had significantly lower levels of DCX in NDEVs compared to healthy controls [105]. On the other hand, Xu et al., using astrocyte-derived EXOs, showed that patients with TRD exhibited significantly elevated levels of two astrocyte markers, GFAP and S100β, as well as CD81, compared with healthy controls. Moreover, inflammatory markers, including IFN-γ, IL-1β, IL-4, IL-6, and TNF-α, were also markedly higher in the TRD group [127]. This study supports the neuroinflammatory hypothesis and the role of astrocyte activation in contributing to TRD. Interestingly, Korlatowicz et al. found that alterations in the specific miRNA profile in lateral habenula (LHb) and medial habenula (MHb) differentiate TRD-like rats from Wistar Han (WIS) rats. The MHb expression of miR-133a, miR-182, and miR-449a was decreased, and the expression of miR-203a, miR-674, and miR-708 was increased in TRD-like rats compared to WIS rats. In the LHb, miR-133a and miR-708 expression were higher, while miR-92a displayed lower expression [128]. Given that miRNAs originating in the CNS can be transported to the periphery via EXOs, and the use of blood-derived EXOs provides a valuable and minimally invasive approach for investigating ongoing molecular alterations within the CNS, this study represents a promising avenue for future research.

EXOs may function as dynamic biomarkers that reflect the response to antidepressant treatment. Exosomal miRNA may predict antidepressant response in patients with MDD. Hung et al. found that patients who achieved remission exhibited lower baseline levels of let-7e, miR-21-5p, miR-145, miR-146a, and miR-155, followed by increased expression after antidepressant treatment compared with the non-remitters [129]. A study utilizing astrocyte-derived EXOs demonstrated that electroconvulsive therapy (ECT) induced a significant reduction in GFAP, S100β and CD81 levels, accompanied by a marked decrease in IFN- γ and IL-4 levels, which correlated with a reduction in the severity of depressive symptoms in TRD patients [127]. Furthermore, analysis of NDEVs from individuals with TRD revealed that ECT increased DCX levels [105]. In addition, recent analysis of exosomal miRNA profiles in patients with recurrent depressive disorder (RDDs) before and after ECT showed that hsa-miR-142-5p, hsa-miR-1908-5p, and hsa-miR-450b-5p may be potential biomarkers for RDD diagnosis and ECT treatment response, especially a substantial proportion of patients with RDD experience TRD [130]. Growing evidence suggests the use of EXOs as biomarkers for pharmacological treatment of DDs, as well as for predicting treatment efficacy. Nevertheless, there is a lack of studies evaluating exosomal profiles in patients with MDD or TRD following ketamine or esketamine treatment. To our knowledge, only a single study has assessed serum exosomal miRNA profiles after esketamine treatment; however, this study was conducted in patients with chronic obstructive pulmonary disease [131]. This highlights a significant need for further investigation.

### 6.2. Exosome-Based Therapeutic Strategies

Emerging evidence emphasizes EVs, particularly EXOs, as promising platforms for both biomarker discovery and therapeutic delivery in DDs. Owing to their endogenous origin, low immunogenicity and efficient BBB penetration, EXOs may function as natural nanocarriers for proteins, peptides, small molecules and enzymes, enabling targeted modulation of molecular pathways implicated in DDs.

Wang et al. fabricated an exosome-functionalized Prussian blue (PB) nanozyme that can self-deliver geniposide (GEN), enabling BBB crossing and synergistic antioxidant therapy. The porous PB carrier exhibits multi-enzyme-like activity, facilitating effective scavenging of accumulated ROS. This antioxidant capacity protects GEN from oxidation within the mildly inflammatory, acidic microenvironment, while GEN synergizes with PB to activate the Nrf2-ARE pathway, thereby improving endogenous oxidative stress defense mechanisms [132]. In basal conditions, kelch-like ECH -associated protein 1 (Keap1) binds to Nrf2, maintaining it in the cell cytoplasm or targeting it for ubiquitin-mediated degradation. Under oxidative stress, excessive ROS induce conformational changes in Keap1, leading to the dissociation of Nrf2, which translocates to the cell nucleus, where it activates the Nrf2/antioxidant response element (ARE) pathway and triggers the expression of several homeostatic genes encoding crucial antioxidant enzymes containing the ARE sequence in their promoters, including heme oxygenase 1 (HO-1), glutathione S-transferase (GST), GPX), CAT and SOD [133]. Notably, recent findings demonstrated that TRD-like rats showed decreased Nrf2 and HO-1 levels in the HP [134]. Moreover, the Nrf2 function may be regulated by the Klotho protein, which acts as an endogenous activator of Nrf2. A recent postmortem study indicated that reduced levels of both Klotho and Nrf2, together with decreased Keap1 levels in the HP and PFC, were associated with suicidal behavior, which is highly prevalent among individuals with TRD [135]. Furthermore, a significant reduction in α-Klotho levels in the PFC of MDD patients was linked to impaired antioxidant defense mechanisms, reflected by decreased CAT and SOD activity [136]. Chen et al. found that Klotho may be involved in mediating the antidepressant effects of low-dose ketamine treatment. Patients with TRD showed elevated Klotho levels after ketamine treatment; interestingly, higher Klotho levels at baseline were associated with diminished antidepressant response during post-infusion follow-up [137]. Collectively, prior studies show that TRD is characterized by significantly increased oxidative stress and neuroinflammation [44]. The Klotho-Keap1-Nrf2-HO-1 signaling pathway constitutes a critical component of the antioxidant defense system and is substantially dysregulated in TRD. Moreover, the nanotherapeutic strategy developed by Wang et al. demonstrates promising efficacy in modulating TRD. Given that oxidative stress is associated with increased ROS generation, Hiu et al. developed a ROS-responsive nanogel loaded with pituitary adenylate cyclase-activating polypeptide (PACAP) and estrogen (E2), sheathed with EXOs, referred to as HA NGs@exosomes [138]. Therapy with HA NGs@exosomes attenuated ROS-induced lipid peroxidation and inflammatory responses, which are strongly implicated in the pathophysiology of TRD [44]. Concurrently, oxidative stress acts as a potent trigger of microglial activation, leading to the release of proinflammatory cytokines r and the establishment of a sustained proinflammatory microenvironment within the CNS. Moreover, recent findings indicate that ketamine, an effective approved treatment for TRD, may attenuate the microglial activation [139]. Notably, a study conducted by Hellmann-Regen et al. showed that minocycline, which inhibits microglial activation, failed to exert antidepressant effects in patients with TRD [140]. Therefore, Lv et al. designed neurologically active celastrol/minocycline encapsulated in macrophage-derived EXO-functionalized PLGA nanoformulations (CMC-EXPL) and evaluated their therapeutic efficacy in a rat model of post-stroke depression. The dual drug molecules loaded EXPL nanoformulations not only exerted significant antidepressant effects but also effectively suppressed neuroinflammation and neuronal damage. Moreover, these nanoformulations inhibited pro-inflammatory M1 microglial polarization while promoting the anti-inflammatory M2 phenotype, which may further contribute to their antidepressant efficacy [141]. Minocycline may possess antidepressant potential in TRD; however, optimizing therapeutic approaches remains necessary, highlighting the need for further research. On the other hand, Yu et al. engineered an alternative approach to modulate microglial activation by designing a delivery system for circular RNA (circDYM) using RVG-modified EVs (RVG-circDYM-EVs). RVG-circDYM-EVs significantly suppressed microglial activation and BBB permeability, while interacting with the transcription factor TAF1 (TATA-box binding protein associated factor 1) to inhibit neuroinflammation, ultimately resulting in the alleviation of depressive-like behaviors [142]. Oxidative stress and neuroinflammation, in turn, influence brain tissue, initiating alterations in neuroplasticity. In this context, Liu et al. designed RVG-modified EXOs engineered to overexpress BDNF (RVG-BDNF-Exos), which modulated the BDNF/TrkB/AKT signaling pathway, improving neuroplasticity [143]. Furthermore, Sig-1R was found to inhibit microglial activation, thereby reducing production of pro-inflammatory cytokines and increasing the expression of neurotrophic factors (e.g., BDNF), ultimately exerting a neuroprotective effect [144]. Guo et al. demonstrated that ketamine activates the mitochondrial protein TAMM41, thereby facilitating the transfer of astrocyte-derived S1R via the TAMM41-cardiolipin- EXOs axis, which was associated with an antidepressant effect. Moreover, the authors established a strategy for producing Sig-1R-enriched EXOs (S1R-EXOs) using human red blood cell-derived EXOs loaded with synthetic S1R mRNA. S1R-EXOs demonstrated antidepressant effects [145].

### 6.3. Regulatory and Translational Challenges for Exosome-Based Approaches in Psychiatry

Due to their endogenous origin, biocompatibility, and ability to encapsulate and deliver a wide range of therapeutic cargo, exosomes are considered promising therapeutic agents and versatile drug delivery platforms. Nevertheless, several important limitations currently restrict their clinical translation.

A key challenge concerns the efficiency, purity, and standardization of exosome isolation and production protocols. Commonly used techniques, including ultracentrifugation, ultrafiltration, size exclusion chromatography, and polymer-based precipitation, typically enrich for extracellular vesicle populations but may co-isolate contaminants such as protein aggregates, lipoproteins, and cellular debris. Moreover, variability in donor cell type, culture conditions, isolation procedures, and quality control measures contributes to substantial heterogeneity in exosome composition and biological activity, thereby limiting reproducibility, cross-study comparability, and reliable potency assessment [91].

Another major issue involves exosome-based drug delivery systems and the efficient and reproducible loading of therapeutic cargo, ranging from small molecules to proteins and nucleic acids. Loading strategies are generally classified as endogenous (pre-secretory) or exogenous (post-isolation) approaches. Endogenous strategies include genetic engineering of donor cells to enable stable and targeted incorporation of therapeutic RNAs or proteins into secreted vesicles, as well as passive incubation of cells with certain small molecules. The former approach offers high specificity and controlled cargo expression but is technically complex, time-consuming, and challenging to scale. The latter is simpler but often associated with limited loading efficiency and reduced control over cargo incorporation.

Exogenous loading methods involve manipulation of isolated exosomes, including electroporation, sonication, freeze–thaw cycles, or passive incubation. These techniques allow direct incorporation of therapeutic agents without modifying donor cells. Although relatively rapid and adaptable, they may compromise membrane integrity, promote cargo aggregation, induce nucleic acid degradation, or result in batch-to-batch variability [92]. Therefore, careful optimization and standardized validation are required to balance loading efficiency with vesicle stability.

Targeting specificity represents another critical limitation. After systemic administration, exosomes may be rapidly cleared by the mononuclear phagocyte system, particularly in the liver, spleen, and lungs, reducing their bioavailability at target sites. Strategies such as surface expression of CD47, PEGylation, biomimetic coating, or optimization of vesicle size and surface properties may reduce immune clearance. However, certain modifications, including PEGylation, may also impair cellular uptake or alter biodistribution. Thus, targeting efficiency must be carefully balanced with preservation of functional uptake.

Effective therapeutic action also depends on cytoplasmic release of cargo following cellular internalization. To enhance endosomal escape, fusogenic peptides (e.g., GALA or H5WYG) can be employed, as they undergo conformational changes in acidic endosomal environments, promoting membrane disruption and cytosolic delivery. pH-sensitive linkers and lipid-based modifications may similarly facilitate controlled intracellular release. Alternative methods such as mild electroporation or saponin treatment can increase membrane permeability; however, these approaches require optimization due to potential risks, including vesicle damage, RNA degradation, aggregation, or inconsistent loading efficiency [91,92].

Targeted delivery is particularly relevant in DDs, where therapeutic efficacy may require crossing the BBB. The BBB is a highly selective physiological barrier that limits the penetration of most therapeutics into the CNS. Surface engineering of exosomes and the development of hybrid nanocarrier systems can enhance CNS targeting while maintaining biocompatibility. For example, the rabies virus glycoprotein (RVG) peptide fused to Lamp2b has been used to facilitate neuronal targeting via interaction with nicotinic acetylcholine receptors. Additionally, ligands targeting transferrin receptors, which are highly expressed on brain endothelial cells, and integrin-binding motifs, such as RGD peptides, can improve brain uptake and biodistribution while reducing off-target accumulation [146].

Immunogenicity remains an additional consideration. Although exosomes are generally regarded as low-immunogenic, residual protein contaminants or incomplete purification may increase immunostimulatory potential. Furthermore, exosomes derived from certain cellular sources may contain immunomodulatory molecules, including MHC proteins, cytokines, or regulatory miRNAs, which could influence host immune responses. Therefore, comprehensive characterization of both exosomal surface markers and cargo is essential to ensure safety and minimize unintended biological effects [91].

Finally, clinical translation is hindered by low yield, product heterogeneity, and the absence of universally accepted potency assays and batch-release criteria. The lack of standardized frameworks for evaluating safety, efficacy, and quality control further complicates regulatory approval and slows clinical implementation. Addressing these challenges will be essential for the successful development of exosome-based therapeutics.

## 7. Integration of Multi-Omics and Artificial Intelligence in Exosome-Based Precision Psychiatry

Multi-omics refers to an integrative analytical framework that combines multiple layers of biological data derived from distinct “omes”, including the genome, transcriptome, proteome, epigenome, microbiome, and metabolome, to enable comprehensive characterization of biological systems. In this context, EVs, particularly EXOs, represent an additional layer of biological complexity owing to their fundamental role in intercellular communication and the targeted transfer of molecular signals between cells and tissues. As stable circulating vesicles carrying a molecular cargo that reflects the functional state of their originating cells, EXOs provide a unique and accessible multi-omics source of information, especially in the CNS, where direct access to brain tissue is limited [147]. Artificial intelligence (AI) comprises computational methods for data-driven prediction and classification. Deep learning (DL), a major AI approach, employs multilayer neural networks to automatically extract hierarchical features from high-dimensional data. AI-based models have demonstrated good performance across biomedical applications, including cell classification, cancer detection, pathological diagnosis and multiple biomarker analysis [148]. However, research on AI in the context of DDs remains limited. A growing body of evidence supports the use of multi-omics approaches to identify the complex, heterogeneous neurobiological mechanisms underlying MDD. For example, a recent study employed plasma exosome transcriptomics, mass spectrometry-based proteomics, and non-targeted metabolomics analyses, demonstrating that differences in metabolites, expressed proteins, genes and miRNAs are associated with the progression of depressive-like behaviors. Rats after traumatic spinal cord injury that developed depression-like behaviors showed alterations in amino acid metabolism and disruptions in several signaling pathways (e.g., endocytosis, TNF, Toll-like receptor, PI3K-Akt pathway) compared to rats that did not develop depressive-like behaviors [149]. On the other hand, Lou et al. used a two-stage cross-scale imaging transcriptomic framework integrating population-level and individual-level multi-omics data to systematically investigate the role of miR-134 in MDD pathophysiology. By combining transcriptomic data, whole-brain functional connectivity, peripheral exosomal miR-134 expression and clinical phenotypes, the authors demonstrated that miR-134-regulated gene networks are closely linked to hierarchical patterns of brain dysconnectivity, particularly along the sensorimotor association axis. Notably, the individual imaging genetics analysis revealed significant associations between reduced exosomal miR-134 expression, altered regional functional connectivity and depressive symptoms severity [150]. To date, few studies have applied a multi-omics approach to investigate DDs; although the single-center prospective observational DetECT study (“Multimodal Biomarkers of ECT in TRD”) has been registered, outcome data are not yet available [151]. However, recent studies increasingly focus on the simultaneous integration of alterations in exosome-derived miRNA expression and accompanying structural brain changes in DDs. Functional neuroimaging studies have shown that pre-treatment activity within the anterior cingulate cortex (ACC), posterior cingulate cortex (PCC), thalamus, insula, and DMN is associated with antidepressant treatment response in MDD. Non-responders exhibit attenuated emotional reactivity and persistent dysfunction within these networks [152]. Conversely, ketamine treatment in TRD patients may normalize the DMN subnetworks and ACC activity [153,154]. Furthermore, patients with TRD showed dysconnectivity in the thalamocortical network [155]. Complementary multimodal approaches integrating exosome-derived microRNA profiling with MRI have identified miR-151a-3p as a molecular correlate of ACC and DMN abnormalities, with normalization observed only in treatment responders [156]. Additionally, in medication-free patients with MDD, reduced cerebral cortex thickness (e.g., in ACC) has been linked to exosomal miR-146a-5p overexpression [157]. Collectively, thalamocortical and corticolimbic circuit alterations may underlie both impaired treatment response and TRD pathophysiology. These findings highlight the potential of integrating exosome-derived miRNA profiling with neuroimaging as a basis for future multi-omics studies elucidating molecular, neural and peripheral alterations underlying TRD. At the same time, ketamine and esketamine have been shown to modulate gene expression in patients with TRD as well as in in vitro models, affecting pathways involved in neuroplasticity, immune regulation, metabolism and glutaminergic signaling, including genes such as glutamate metabotropic receptor 2 (*GRM2*) and NMDAR subunit 2D (*GRIN2D*) [158]. Prior to ketamine administration, responders exhibited upregulation of the *GRM2* and *GRIN2D* compared with non-responders [159]. Experimental evidence further suggests that downregulation of miR-342-3p in epilepsy models may increase *GRM2* expression, indicating a potential mechanism underlying a positive therapeutic response in TRD [160]. Taken together, these findings suggest a framework for identifying exosome-derived miRNAs targeting *GRM2* and *GRIN2D,* which could serve as predictive biomarkers for ketamine response. For instance, low exosomal expression of miR-342-3p may be associated with upregulation of *GRM2*, potentially predicting a beneficial response to ketamine therapy. Further studies employing larger, multi-omics approaches are required to validate these findings and elucidate the mechanistic underpinnings of ketamine responsiveness in TRD.

All original studies included in Table 2 included exosomes characterized according to the MISEV criteria, including analysis of particle size distribution by NTA/DLS, assessment of vesicle morphology by TEM, and identification of protein markers specific to this category (see Table 2).

## 8. Conclusions and Future Perspectives

The prevalence of DDs continues to rise globally. Despite the availability of a wide range of pharmacological interventions, approximately two-thirds of patients experience relapse or fail to achieve an adequate response to standard treatments. It is currently estimated that at least 30% of individuals with DDs meet the definition of TRD [93]. Furthermore, recent studies indicate that nearly half of patients with MDD meet TRD criteria, reflecting a growing clinical burden [163]. Nevertheless, both diagnostic and therapeutic approaches for TRD remain limited. A clinical diagnosis of TRD typically requires the absence of a clinically meaningful response to at least two adequate antidepressant treatment trials. Considering that an adequate trial is generally defined as 6–8 weeks of treatment, a minimum of 12–16 weeks is necessary to confirm TRD [164]. In real-world population-based cohorts, the interval between initiation of first-line therapy and escalation to third-line treatment (TRD) often exceeds 12 months, suggesting that in practice, TRD diagnosis may be considerably delayed relative to formal criteria [165]. This highlights the need to identify innovative disease biomarkers that could accelerate diagnosis. A growing body of research confirms the effectiveness of exosome-derived biomarkers. EXOs are nanoscale extracellular vesicles capable of transporting a diverse molecular cargo, including proteins, cytokines, and miRNAs, which can reflect CNS alterations associated with TRD (Figure 2). Consequently, exosomal profiling may provide sensitive and specific indicators of treatment resistance. Moreover, integrating exosome-derived data with neuroimaging findings and multi-layered omics methods encompassing the genome, transcriptome, proteome, epigenome, microbiome, and metabolome could facilitate the development of advanced multi-omics platforms, enabling a systemic characterization of the molecular, neuronal, and peripheral changes underlying TRD. Collectively, these insights provide a framework to guide future research toward the development of reliable, advanced methodologies for diagnosing TRD.

Ketamine is a drug approved for the treatment of TRD; however, a subset of patients fails to respond to its administration. Given the high financial cost and considerable clinical implications associated with ketamine therapy, identifying predictors of response is a high priority. Recent studies have explored the utility of exosomal miRNAs (let-7e, miR-21-5p, miR-145, miR-146a, and miR-155) as potential biomarkers for differentiating responders from non-responders to conventional antidepressants (Table 2, Figure 3). Yet, investigations specifically exploring exosome-based predictors of ketamine efficacy remain limited, representing an important avenue for future research aimed at optimizing individualized treatment strategies in TRD. The increasing prevalence of TRD underscores the need to explore alternative therapeutic strategies that extend beyond the monoaminergic hypothesis. In this context, EXOs represent a potential therapeutic approach for DDs. They may be employed either as interventions using EXOs isolated from stem cells or through engineered approaches that provide specialized nanocarriers. Preclinical studies have demonstrated that exosome-based therapies modulate pathways implicated in the pathophysiology of TRD, including neuroplasticity, neuroinflammation, and oxidative stress (Table 1). However, current evidence is largely limited to preclinical studies, with a notable absence of reports of negative or null findings, highlighting the need for further research, particularly well-designed clinical trials. Furthermore, one of the major factors limiting the application of EXOs in both the diagnosis and treatment of DDs remains the inaccuracy of current exosome isolation and detection methods. In addition, variability in biological sources and the lack of standardized protocols further constrain the potential utility of EXOs. To date, no studies have investigated the isolation of EXOs from saliva or urine in patients with DDs, and research examining potential differences in the biophysical characteristics of EXOs derived from blood, cerebrospinal fluid, saliva, and urine is still limited. Moreover, most studies focus on a restricted subset of miRNAs or proteins, and the comprehensive molecular landscape of exosomes, which carry diverse biomolecules from multiple cell types, remains poorly characterized. This limits their potential for identifying reliable biomarkers of DDs.

Notably, there is a lack of studies evaluating both the safety of exosomes and their associated adverse effects. While short-term safety profiles were generally favorable, comprehensive longitudinal studies are needed to assess the long-term impact of exosome treatment on aspects such as potential oncogenic risk. The immunogenic potential of exosomes warrants careful consideration. Although exosomes themselves are considered low-immunogenic, their cargo or contaminants resulting from insufficient purification methods may induce immune activation. Prunevieille et al. demonstrated that allogeneic exosomes delivered in an inflammatory environment activate T cells in vivo and sensitize mice to alloantigens [166]. This is particularly relevant in the context of DDs, where inflammation plays a key role. Accordingly, this raises the question of whether increased exosome immunogenicity may compromise their therapeutic efficacy. Furthermore, various cells and tissues may uptake exosomes, complicating the assessment of their biodistribution and therapeutic efficacy. It highlights the need for advanced methodologies to monitor their intracellular trafficking and elucidate mechanisms of action. Additionally, the safety of using exosomes with other pharmaceuticals should be investigated. Gabaran et al. demonstrated that pharmacological agents may modulate exosome functionality, including impairing their cellular uptake [94]. However, the effect of antidepressants such as SSRIs, SNRIs, TCAs, or ketamine on exosome pharmacokinetics has not yet been investigated. It highlights a promising avenue for future research, particularly focusing on the therapeutic and clinical implications of exosomes in DDs.

In conclusion, given the promising potential of exosome-based approaches, further studies are required to elucidate their role in TRD. Key research priorities include establishing standardized, high-quality methods for exosome isolation, characterization, and multi-omics profiling to enable reliable biomarker discovery and clinical translation in TRD. Rigorous evaluation of safety, immunogenicity, pharmacokinetics and interactions between drugs (particularly antidepressants) and exosomes is equally important. These priorities may be addressed by designing longitudinal studies or combination-therapy investigations. Finally, well-designed clinical trials are needed to validate exosome-based diagnostics and therapeutics and to identify treatment response predictors, including response to ketamine.

## Figures and Tables

**Table 1:** Summary of preclinical studies on exosome-derived therapies: effects on depressive-like behaviors and molecular pathways.

*Species/Strain*	*Model of Depression/Treatment*	*Results—Behavioral Tests*	*Samples*	*Methods*	*Results*	*References*
**Male C57BL/6J mice (8–10 weeks old)**	Chronic unpredictable mild stress (CUMS; 6 weeks) Oligodendrocyte-derived exosomes (ODEXs)-containing SIRT2 (100 μL of ODEXs (0.5 μg/μL)/equal volume of PBS; 2 weeks, every 3 days; i.v.)	↑ Sucrose consumption in the sucrose preference test ↓ Immobility time in the forced swim test ↔ Total distance traveled, ↑ time spent in center area in the open field test	Hippocampus (HP)	Exosome isolation: polymer-based precipitation + anti-CNPase immunoaffinity Exosome characterization: TEM, NTA, markers (CD9, CD63, Alix) Western Blot RT-PCR Immunostaining	↑ DCX+ and Nestin+ cells ↑ PSD95, SYP ↑ synapse density ↑ SIRT2 ↑ p-AKT, p-GSK-3β	[111]
**Male C57BL/6 mice (7 weeks old)**	Lipopolysaccharide (LPS)-induced depression (1 mg/kg, i.p; 7 days) Bone marrow mesenchymal stem cell (BMSC)-derived exosomes (200 ul 1.4 × 10^11^/mL; 2 weeks-every 3 days; i.v.)	↑ Total movement distance, the open field test ↓ Immobility time in the tail suspension test and forced swim test ↓ Latency to eat in novelty suppressed feeding test	Hippocampus (HP) Prefrontal cortex (PFC) Serum	Exosome isolation: Polymer-based precipitation Exosome characterization: TEM, NTA, markers (Alix, HSP70) Western Blot qRT-PCR Immunostaining Colorimetric analysis	↓ IL-1β, IL-6, TNF-α in HP and PFC ↑ IL-10 in HP and PFC ↓ Iba-1, GFAP in HP and PFC ↓ MDA, NO in HP and serum ↑ SOD, T-AOC in HP and serum ↑ DCX+ cells in HP	[112]
**Male Kunming mice (30–50 g)**	Chronic unpredictable mild stress (CUMS; 52 days) DPA-treated human umbilical cord mesenchymal stem cells (hUCMSCs)-derived exosomes (10 mg/kg; 1 week-every 3 days; i.p.)	↑ Total distance traveled, number of rearing, entries into the central zone in the open field test ↓ Immobility time in the tail suspension test ↑ Entry number into and the time spent on open arms in the elevated plus maze test	Hippocampus (HP) Prefrontal cortex (PFC) Serum	Exosome isolation: ultracentrifugation Exosome characterization: TEM, NTA, markers (CD9, TSG101) Western Blot ELISA qRT-PCR Immunostaining HPLC-ECD Flow Cytometry	↑ 5-HT, DA concentrations in HP ↓ IL-1β, TNF-α in HP, PFC and serum ↑ IL-10 in HP, PFC and serum ↓ Iba-1, iNOS in HP and PFC ↓ MyD88, TRAF6, NF-κB P65 in HP	[113]
**Male BALB/c mice (18–22 g)**	Chronic mild stress (CMS; 30 days) NK cell-derived exosomes from unstressed mice/stressed mice (66.42 μg; one time; i.v.)	↓ Immobility time in the forced swim test and the tail suspension test ↑ Horizontal movements, vertical movements and exercise time in the open field test	Hippocampus (HP) Hippocampal astrocytes Primary astrocytes-whole brain	Exosome isolation: centrifugation Exosome characterization: TEM, NTA, markers (CD81, CD63) ELISA Immunostaining Western Blot qRT-PCR	Unstressed mice: ↓ IL-1β, IL-6, TNF-α in HP and astrocytes ↑ 5-HT, DA, NE in HP NK exosomes (↑ miR-207 expression) → ↓ Tril/NF-κB → ↓ IL-1β, IL-6, TNF-α in primary astrocytes Stressed mice: antidepressant effect NK exosomes (↓ miR-207 expression)	[114]
**Male C57BL/6 J mice (4–5-weeks old)**	Chronic restraint stress (CRS; 28 days) Adipose-derived mesenchymal stem cell exosomes (ADSC-Exos; 3.0 × 10^7^ EVs/mouse/day for 14 days; i.n.)	↓ Immobility time in the forced swim test and the tail suspension test ↑ Total distance moved and time spent in the center in the open field test ↑ Frequency of entering the open arms, time spent in the open arms in the elevated plus maze ↑ The discrimination index in novel object recognition test	Hippocampus (HP) Serum	Exosome isolation: ultracentrifugation Exosome characterization: TEM, NTA, markers (CD81,HSP70, TSG101,calnexin) Western Blot ELISA IF HE	↓ Bax/Bcl-2 ratio, caspase-3 levels in HP ↑ p-AMPK/AMPK, LC3BII/I ratio in HP ↓ p-mTOR/mTOR ratio, P62 protein expression in HP ↑ autophagosomes in HP ↓ NLRP3, ASC, IL-1β, and caspase-1 protein expression in HP ↓ IL-1β, IL-6, TNF-α in serum ↑ IL-10 in serum ↓ Iba1+ cells, ↑ GFAP+ cells in HP	[115]
**SPF-grade SD pregnant rats (250–300 g); SPF-grade SD female rats (8 weeks old; 300–350 g; n = 32)**	Chronic unpredictable mild stress (CUMS; 21 days) + ovariectomy (OVX) Neural stem cell-derived exosomes (NSCs-Exo; 30 μg/rat in 200 μL PBS; 2 weeks; i.c.v.) + electro-acupuncture treatment (EA; 28 days)	n.a.	Hippocampus (HP)	Exosome isolation: ultracentrifugation Exosome characterization: TEM, NTA, markers (Alix, CD9) Western Blot IF	↑ NeuN+ cells, Nestin+ cells ↑ AMPK, p-AMPK, PGC-1α, NRF1, and TFAM ↑ PSD95, SYN, and GAP43	[110]
**Male Sprague Dawley rats (100 ± 12 g; 6–8 weeks old)**	Corticosterone-induced depression (CORT, 40 mg/kg; s.c.) Bone marrow mesenchymal stem cells (BMSCs)-derived exosomes (100 μg exosomes dissolved in 1 mL PBS; i.v.)	↑ Sucrose consumption in the sucrose preference test ↑ Horizontal movements in the open field test	Hippocampus (HP) Serum	Exosome isolation: ultracentrifugation Exosome characterization: TEM, NTA, markers (Alix, TSG101, CD63) qRT-PCR ELISA Western Blot	↑ miR-26a expression in HP ↑ SOD level in serum and HP ↓ MDA, LDH, TNF-α and IL-1β levels in serum and HP ↓ Bax, ↑ Bcl-2 in HP	[116]
**Male albino rats (Rattus norvegicus 120–150 g)**	Doxorubicin (DOX)-induced dyskinesia and anxiety (2.5 mg/kg/day; 9 days; i.p.) Bone marrow mesenchymal stem cells (BM-MSCs) derived exosomes (1.5 mL/kg-exosomes 10–20 µg/mL every week for three successive weeks; i.p.)	↑ Number of crossed peripheral squares, rearing frequency, time spent in central squares in open field maze ↑ Time spent in the open arms, ↓ time spent in the closed arms in the elevated plus maze test	Cortex Serum	Exosome isolation: centrifugation + TCA-based precipitation Exosome characterization: TEM qRT-PCR ELISA	↔ MDA in serum ↓ JNK, NF-κB, p38 ↑ Erk-1	[117]

Abbreviations: i.v.—intravenous; i.p.—intraperitoneally; s.c.—subcutaneously; i.n.—intranasally; i.c.v.—intracerebroventricular; (↑)—increase while; (↓)—decrease; (↔)—no changes; (→)—indicates a sequentiual relationship between processes; n.a.—not applicable.

**Table 2:** Association between miRNAs and TRD, MDD and RDD. Summary of clinical and preclinical studies.

*Disorder*	*Source/Methods*	*miRNA*	*Direction of Change*	*Molecular Targets/Pathways*	*Clinical Implication*	*References*
**TRD**	Plasma Exosome isolation: spin column-based membrane affinity Exosome characterization: TEM, NTA, markers (CD9, CD81)	miR-335-5p	**↑**	n.a.	Biomarker	[25]
		miR-1292-3p	**↓**	n.a.		
**TRD-like phenotyphe**	Medial Habenula (MHb)	miR-133a miR-182 miR-449a	**↓**	n.a.	Biomarker	[128]
		miR-203a miR-674 miR-708	**↑**	n.a.		
	Lateral Habenula (LHb)	miR-133a miR-708	**↑**	n.a.		
		miR-92a	**↓**	n.a.		
**MDD**	Serum Exosome isolation: ultracentrifugation Exosome characterization: TEM, NTA, markers (CD9, CD63)	miR-9-5p	**↑**	SOCS2/JAK/STAT3 **↑** IL-1β, IL-6, and TNF-α	Potential therapeutic target	[161]
**MDD**	Serum Exosome isolation: density gradient centrifugation Exosome characterization: TEM, DLS, markers (syntenin, TSG101, CD81)	miR-139-5p	**↑**	BDNF	Biomarker/Potential therapeutic target	[162]
**MDD**	Serum Exosome isolation: Polymer-based precipitation	let-7e miR-21-5p miR-223 miR-146a	**↑**	n.a.	Predictor of antidepressant response	[129]
**Rat model of depression-like behavior**	BMSCs-derived Exosome isolation: ultracentrifugation Exosome characterization: TEM, NTA, markers (Alix, TSG101, CD63)	miR-26a	**↑**	SOD MDA, LDH, TNF-α and IL-1β	Potential therapeutic target	[116]
**MDD**	Plasma Exosome isolation: precipitation + purification (Exosome Purification Filter) Exosome characterization: TEM, NTA, markers (CD9, CD63, CD81)	miR-151a-3p	**↑**	n.a.	Predictor of antidepressant response	[156]
**RDD**	Serum Exosome isolation: total exosome isolation reagent-based precipitation Exosome characterization: TEM, NTA, markers (CD9, CD63)	hsa-miR-142-5p	↓	CRP, NLR, IL-1α	Predictor of antidepressant response	[130]
		hsa-miR-450b-5p	↓	ZNF611		
		hsa-miR-1908-5p	↑	NAP1L4		

Abbreviations: (↑)—increase while; (↓)—decrease; n.a.—not applicable.

## Data Availability

This review article relies exclusively on previously published data, fully cited in the bibliography. The original datasets used in the cited studies were not accessed or analyzed by the authors.
